# Supplementary material for: A Comprehensive Assessment of Ultraviolet-Radiation-Induced Mutations in Flammulina filiformis Using Whole-Genome Resequencing
Source: J Fungi (Basel). 2024 Mar 20;10(3):228. doi: 10.3390/jof10030228 (PMC10971301; doi:10.3390/jof10030228)
Supplement: Supplementary file 1 [file jof-10-00228-s001.zip › Supplementary Material S8/KEGG annotation/out/64381550635650.os/KO/out_map/map04011.html]

KEGG PATHWAY: MAPK signaling pathway - yeast - Reference pathway


|  |  |
| --- | --- |
| **MAPK signaling pathway - yeast - Reference pathway** |  |

[
Pathway menu
| Organism menu
| Pathway entry
| Show description
| User data mapping
]

The S. cerevisiae genome encodes multiple MAP kinase orthologs. One (Fus3) mediates cellular response to peptide pheromones. Another (Kss1) permits adjustment to nutrient limiting conditions. A third (Hog1) is necessary for survival under hyperosmotic conditions. A fourth (Slt2/Mpk1) is required for repair of injuries to the cell wall. As in mammalian cells, these pathways consist of a conserved module in which three kinases phosphorylate each other in sequence. The MAPK is phosphorylated by the MAPK/ERK kinase (MEK), which is itself phosphorylated by a MEK kinase (MEKK).

|  |  |
| --- | --- |
| Reference pathway | 100% |
